# Simultaneous Dengue and Chikungunya Coinfection in Endemic Area in Brazil: Clinical Presentation and Implications for Public Health

**DOI:** 10.21203/rs.3.rs-4277561/v1

**Published:** 2024-04-22

**Authors:** Paula Marilia Afonso Torres, Debora Glenda Lima de La Roque, Lucca Rocha Policastro, Lilian Beatriz Moreira Oliveira Chagas, Denise Bergamaschi Giomo, Danielle Cristina Dacanal Gentil, Vagner Fonseca, Maria Carolina Elias, Sandra Coccuzzo Sampaio, Marta Giovanetti, Benedito Antônio Lopes Fonseca, Rodrigo Tocantins Calado, Luiz Carlos Alcantara, Dimas Tadeu Covas, Flávia Leite Souza Santos, Simone Kashima, Luzia Márcia Romanholi Passos

**Affiliations:** Divisão de Vigilância Epidemiológica/Departamento de Vigilância em Saúde; Blood Center of Ribeirão Preto; Medical School of Ribeirão Preto; Medical School of Ribeirão Preto; Divisão de Vigilância Epidemiológica/Departamento de Vigilância em Saúde; Divisão de Vigilância Epidemiológica/Departamento de Vigilância em Saúde; Pan American Health Organization (PAHO)/World Health Organization (WHO); Butantan Institute; Butantan Institute; Fundação Oswaldo Cruz; Medical School of Ribeirão Preto; Medical School of Ribeirão Preto; Fundação Oswaldo Cruz; Medical School of Ribeirão Preto; Blood Center of Ribeirão Preto; Blood Center of Ribeirão Preto; Divisão de Vigilância Epidemiológica/Departamento de Vigilância em Saúde

**Keywords:** Dengue virus, Chikungunya virus, Co-infection, Clinical studies, Molecular Characterization, Phylogenetics

## Abstract

**Background:**

Dengue virus (DENV) and Chikungunya virus (CHIKV) pose significant public health threats in Brazil, where favorable conditions facilitated the proliferation of *Aedes* mosquitoes. Since the mid-1980s, Brazil has experienced annual outbreaks of DENV, with recent increases in confirmed cases. In addition, CHIKV, which was first reported in 2014, has spread across the country. The concurrent presence of these viruses has triggered public health alerts in endemic regions, underscoring the complexity of managing vector-borne diseases.

**Case Presentation:**

This report details a case of simultaneous DENV and CHIKV infections. A 77-year-old female patient who has diabetes and arrhythmia exhibited symptoms including fever, myalgia, and severe arthralgia. Laboratory tests confirmed the coinfection through RNA detection. The patient received supportive care, showed gradual improvement, and was eventually discharged.

**Conclusions:**

Coinfection with DENV and CHIKV cases reported here developed with mild outcomes. However, one of the patients did not recover from the arthralgia after presenting diagnostic challenges, which underscores the need for accurate differentiation to manage symptoms effectively. The reported cases, amidst increasing DENV outbreaks, highlight the urgency for preparedness in the healthcare system. The Ribeirão Preto region’s endemicity for DENV, coupled with the rising incidence of CHIKV, emphasizes the evolving landscape of arbovirus transmission. Studies on *Aedes* mosquitoes suggest potential implications for human infection dynamics, warranting further investigation into arbovirus transmission efficacy and coinfection dynamics.

## Background

Dengue virus (DENV) and Chikungunya virus (CHIKV) are arboviruses transmitted by the *Aedes aegypti* mosquito and belong to the *Flavivirus* and *Alphaviruses* genera, respectively [1, 2]. As a tropical country, Brazil provides favorable conditions for the expansion and proliferation of *Aedes* mosquitoes. This condition allows these arboviruses to pose a significant threat to public health. Although Aedes mosquitoes were eradicated from Brazil in 1973, they returned in 1976–1977 due to their presence in neighboring countries [3]. They are now spread throughout the entire territory of Brazil, a phenomenon driven not only by the climate but also by population migration and intensive use of non-biodegradable materials [4].

DENV is classified into four serotypes: DENV-1 to DENV-4; each serotype is further divided into genotypes with up to a 6% genetic divergence [5]. The first outbreak in the Brazilian territory after the reemergence of Aedes mosquitoes was caused by DENV-1 genotype V in Rio de Janeiro, a state located in the southeast portion of Brazil [4, 6]. Since then, Brazil has experienced annual outbreaks, continuing up to the present, with introductions of new serotypes and genotypes. In the first two months of 2024, the number of confirmed cases has been alarmingly high compared to previous years across several states. These data have triggered a public health alert in many regions of Brazil. For this reason, the Ministry of Health established an emergency committee to coordinate a nationwide response and assist symptomatic individuals.

Genetic variability of CHIKV classifies it into four distinct genotypes—West African, Asian, East/Central/South African (ECSA), and Indian Ocean Lineage ([7]. In 2014, CHIKV was first reported in the northern region of Brazil, along the border with French Guiana. Since its introduction, CHIKV, while less prevalent than dengue, has had a significant impact on public health as it spread throughout the country [8] [9]. Until 2017, the most prevalent Brazilian region was the Northeast, but since then, many cases have arisen, mainly in the Southeast region.

Ribeirão Preto is an endemic area for DENV, and CHIKV has always been detected in inexpressive numbers, being exclusively imported cases [10]. Nevertheless, in 2023, a significant increase in the number of CHIKV autochthonous cases was [11]. DENV and CHIKV co-circulation are challenging for health agencies, as they highlight the complexities of managing vector-borne diseases in endemic regions.

Here, we report a case of simultaneous DENV and CHIKV infections, underscoring the importance of accurate differential diagnosis to manage coinfection symptoms and the viral genomic aspects.

## Case presentation

A 77-year-old female patient had her plasma sample collected during the 47th epidemiological week of 2023. DENV-NS1 antigen and CHIKV-PCR were positive (cycle threshold (Ct) value of 18.6). The onset of the symptoms was November 18, 2023 (Day 1). No other positive cases were reported in the family.

Medical History: On Day 1, the patient presented with fever, myalgia, and severe arthralgia. On Day 3, laboratory tests for dengue revealed a positive NS1 ELISA and a non-detectable RT-PCR, while for Chikungunya RT-PCR was detectable, and IgM-ELISA was reactive. The coinfection was confirmed through Dengue NS1 antigen and Real-Time PCR for CHIKV [12]. RT-PCR for DENV was negative.

Three days after the symptom’s onset, the patient sought medical attention, presenting with headache, myalgia, fever, arthralgia, and rash. Additionally, nausea and loss of appetite were reported, but no swelling was observed. The patient’s medical history included type 2 diabetes and arrhythmia, which made her part of a risk group for adverse outcomes. Furthermore, the initial blood count showed hemoconcentration in accordance with the upper reference limit for the age group (41 ± 6%) (Table 1). Therefore, treatment with intravenous saline solution was immediately started, and she was closely monitored for signs of severe Dengue. Joint pain and myalgia were relieved with analgesics (dipyrone and paracetamol), without the use of anti-inflammatories. Twenty-four days after symptoms onset, the patient experienced cervical pain radiating to the right shoulder, along with pain and swelling in the feet, which lasted for 15 days; swelling of the right wrist was also noted. Two months after the initial presentation, the patient was still suffering from persistent myalgia.

Despite the hemoconcentration typical of Dengue, the blood count did not reveal thrombocytopenia. The white blood cell count was within normal limits but showed lymphopenia, which can be found in dengue and chikungunya infections. Liver enzymes were only slightly elevated, with AST levels higher than ALT levels, as commonly seen in dengue fever (Table 1). No significant changes were observed in biochemical parameters, such as urea, creatinine, sodium, and potassium.

Previous studies demonstrated that elevation of aminotransferases can be attributed to the direct effect of the dengue virus on liver cells, as well as the immune response to the infection [13]. Significantly elevated levels of AST and ALT are often associated with more severe dengue manifestations and can serve as a marker for the severity of the infection [13]

Treatment and Outcome: Over the following two weeks, the patient’s clinical condition gradually improved. Subsequently, she was discharged with instructions for adequate rest and scheduled for follow-up appointments to ensure continued recovery.

## Discussion and conclusions

In this study, we outline two cases of simultaneous infection with DENV and CHIKV in Brazil, a new finding in a region previously undocumented for such coinfections. Despite the rarity of DENV-CHIKV coinfection discussions, most are from India, where the endemicity for both viruses is high, and a coinfected rate of 9.5% was reported [14, 15,16].

The detection of DENV and CHIKV coinfections in December 2023, ahead of the anticipated epidemic season of April to June 2024, underscores an urgent need for the healthcare system to adapt to this evolving threat. These cases were identified in the Ribeirão Preto region, an area with a significant history of dengue fever outbreaks, with annual confirmed cases reaching between 12,000 and 17,000 in recent years. Interestingly, CHIKV incidents belonging to imported origin have remained relatively low, according to the region’s health bulletin, over the past five years (ranging from 0 to 8 cases per year) [11]. However, a notable increase in autochthonous cases was observed towards the end of 2023, November and December, the peak periods. This critical shift in the epidemiological landscape requires vigilant public health responses.

The spread of arboviruses is intricately linked to various factors, mainly climate factors such as rainy periods and extreme heat, which favor the proliferation of *Aedes aegypti*, the common vector for both DENV and CHIKV. Peculiarly, *Aedes aegypti* mosquitoes have been extensively studied, revealing that this mosquito species can harbor hosts and vectors for both viruses *in vitro* settings [16,17]. Lin and colleagues (2023) further demonstrated that simultaneous infection with DENV and Zika virus in *Aedes aegypti* could intensify viral replication within the mosquito [18]. This enhanced replication capacity, alongside the mosquito’s ability to transmit multiple viruses, raises critical questions regarding the potential for human infection resulting from successive exposures to mosquitoes carrying DENV, CHIKV, or both.

Considering the shared transmission vector for DENV and CHIKV, the dynamics of these coinfections among humans justify further investigation to understand the full scope of arbovirus transmission efficacy [19]. This case was detected in the same areas where most CHIKV-confirmed cases have been identified. The discrepancy between the results of serology and RT-PCR is explained. Early recognition and appropriate medical care are essential for a favorable patient outcome, although many clinical symptoms overlap between these viruses.

The approval of the DENV vaccine, QDenga (Takeda, Japan), in March 2023, with the campaign launched in February 2024 focusing on specific age groups and priority regions, represents a significant advancement in dengue prevention. This vaccine is an attenuated virus with an efficacy of 72.7% (López-Medina et al., 2022). However, one of the main critical challenges of managing dengue’s four serotypes: DENV-1, DENV-2, DENV-3, and DENV-4; once the infection with one serotype does not provide immunity against the others.

This challenge is exacerbated by the increasing number of autochthonous CHIKV cases in our region. Notably, CHIKV infection is already endemic in many other regions of Brazil, such as the Northeast, leading to coinfections that can complicate the diagnosis and management of patients due to overlapping clinical symptoms. Moreover, numerous questions remain unanswered regarding whether dual infection could result in more severe disease and complications. Additionally, the implementation of discriminatory diagnostics for DENV and CHIKV is currently lacking in Brazil’s public healthcare systems.

Our findings reinforce the critical need for comprehensive research into the dynamics of coinfection, immune responses, and the interactions between these viruses, aspects that remain insufficiently understood. There is a clear imperative need for robust surveillance systems, advanced diagnostic tools, effective vector management strategies, and public health education campaigns to mitigate the impact of these arboviral infections. Understanding and addressing the nuances of coinfection will be crucial in formulating effective responses to these public health challenges.

## Figures and Tables

**Table 1.** Blood count parameters and biochemical parameters.

Parameters	Case Report	
	Day 3	Day 24^th^
Hemoglobin (g/dL)	14.4	13.9
Hematocrit (%)	47.7	44
Platelets count (x10^9^/L)	156.8	224
WBC (x10^9^/L)	6.6	8.8
Lymphocyte (%)	10.6	21.5
Neutrophils (%)	80.2	68.6
Eosinophils (%)	0.1	0.6
Basophils (%)	0.8	1
Monocytes (%)	8.4	8.4
Use the “Insert Citation” button to add citations to this document.	88	ND
AST (U/L)		
ALT (U/L)	53	ND
Urea (mg/dL)	44	ND
Creatinine (mg/dL)	0.70	ND
Sodium (mmol/L)	146	ND
Potassium (meq/L)	4.7	ND

Abbreviations: WBC; White Blood Cells; AST: Aspartate aminotransferase; U/L: units per liter; ALT: Alanine aminotransferase; ND: non-determined.
